# HARNESSING NETWORKS IN TRANSLATIONAL GEROSCIENCE RESEARCH

**DOI:** 10.1093/geroni/igad104.1074

**Published:** 2023-12-21

**Authors:** Jamie Justice

**Affiliations:** Wake Forest University School of Medicine, Winston-Salem, North Carolina, United States

## Abstract

Understanding and addressing age-related chronic diseases and disability requires a “root cause” approach: to look beyond the individual diseases to biological aging as the shared driver of multiple conditions. This goal is the foundation of an emerging discipline, geroscience. Geroscience has fostered new research on the basic biology of aging with rapid development of biomarkers of biological age, and therapeutics that target aging biology advancing to clinical trials. This presentation will provide a translational framework for early-career investigators who seek to evaluate biological processes underlying aging, and test promising interventions targeting these processes from animal models to clinical trials. This includes: 1) developing outcomes and biomarker frameworks for aging outcomes trials, and 2) establishing functional consequences of cellular senescence in older adults; and 3) creating opportunities to grow a diverse inter-disciplinary research program in geroscience through professional organizations, scientific networks, and NIA-supported research centers.

